# Detection of *Wolbachia* in the Tick *Ixodes ricinus* is Due to the Presence of the Hymenoptera Endoparasitoid *Ixodiphagus hookeri*

**DOI:** 10.1371/journal.pone.0030692

**Published:** 2012-01-26

**Authors:** Olivier Plantard, Agnès Bouju-Albert, Marie-Astrid Malard, Axelle Hermouet, Gilles Capron, Hélène Verheyden

**Affiliations:** 1 Biology, Epidemiology and Risk Analysis in Animal Health, (BioEpAR), INRA, UMR 1300, Nantes, France; 2 LUNAM Université, Oniris, Nantes, France; 3 ONCFS, DIR Poitou-Charentes-Limousin, Paris, France; 4 Comportement et Ecologie de la Faune Sauvage (CEFS), INRA, UR 035, Castanet-Tolosan, France; The Johns Hopkins University School of Medicine, United States of America

## Abstract

The identification of micro-organisms carried by ticks is an important issue for human and animal health. In addition to their role as pathogen vectors, ticks are also the hosts for symbiotic bacteria whose impact on tick biology is poorly known. Among these, the bacterium *Wolbachia pipientis* has already been reported associated with *Ixodes ricinus* and other tick species. However, the origins of *Wolbachia* in ticks and their consequences on tick biology (known to be very diverse in invertebrates, ranging from nutritional symbionts in nematodes to reproductive manipulators in insects) are unknown. Here we report that the endoparasitoid wasp *Ixodiphagus hookeri* (Hymenoptera, Chalcidoidea, Encyrtidae) – strictly associated with ticks for their development - is infested at almost 100% prevalence by a *W. pipientis* strain belonging to a *Wolbachia* supergroup that has already been reported as associated with other hymenopteran parasitoids. In a natural population of *I. ricinus* that suffers high parasitism rates due to *I. hookeri*, we used specific PCR primers for both hymenopteran and *W. pipientis* gene fragments to show that all unfed tick nymphs parasitized by *I. hookeri* also harbored *Wolbachia*, while unparasitized ticks were *Wolbachia*-free. We demonstrated experimentally that unfed nymphs obtained from larvae exposed to *I. hookeri* while gorging on their vertebrate host also harbor *Wolbachia*. We hypothesize that previous studies that have reported *W. pipientis* in ticks are due to the cryptic presence of the endoparasitoid wasp *I. hookeri*. This association has remained hidden until now because parasitoids within ticks cannot be detected until engorgement of the nymphs brings the wasp eggs out of diapause. Finally, we discuss the consequences of this finding for our understanding of the tick microbiome, and their possible role in horizontal gene transfer among pathogenic and symbiotic bacteria.

## Introduction

As ticks are considered to be second only to mosquitoes as vectors of human infectious diseases in the world, the identification of micro-organisms carried by ticks is an important issue for human or animal health [1]. Among arthropod vectors, ticks harbor the largest diversity of micro-organisms, ranging from viruses (*i.e.* arboviruses such as Tick Borne Encephalitis…), prokaryotes (including spirochaetes such as *Borrelia burgdorferi*) and eukaryotes (including Apicomplexa - such as *Babesia* spp. or *Theileria* spp. – or even metazoans - such as some filarial nematodes -) [2]. This large diversity of micro-organisms may be related to the fact that all three stages (larvae, nymphs and adults) take a blood meal over several days, ingesting a large volume of blood [3], and that a wide range of different vertebrate host species is used for the blood meal (at least for the most common species in Europe or in North-America – *i.e*. *Ixodes ricinus* and *I. scapularis* respectively). Moreover, because of the long life cycle of ticks (usually 2 to 3 years for species living in temperate areas), these arthropods are reservoirs for pathogens (especially for vertically transmitted pathogens, i.e. trans-ovarial – transmission)in addition to vertebrate primary hosts, facilitating micro-organism persistence through unfavorable seasons [4].

However, ticks do not only harbor pathogens; they also host symbiotic bacteria whose effects on tick fitness and on vertebrate health are poorly known. For example, among *Rickettsia* spp., several species that are commonly found associated with ticks have never been found associated with disease [5], [6]. While *Coxiella burnetti* is known as the etiological agent of the Q-Fever responsible for recent outbreaks in Europe, an unnamed *Coxiella* species has been found in 100% of the individuals of *Amblyomma americanum* [7]. Antibiotic treatment of these ticks resulted in reduced reproductive fitness, suggesting that the *Coxiella* sp. is a primary endosymbiont [8]. *Midichloria mitochondrii* is an endosymbiotic alpha-proteobacterium found in the ovarian tissues of almost all *Ixodes ricinus* females from natural populations [9], [10]. This bacterium has a unique intramitochondrial lifestyle [11], [12], [13] but its consequences on tick fitness or reproduction remain unknown [13]. More recently, an obligate intracellular gamma-proteobacterium (*Diplorickettsia massiliensis*) has been isolated from *I. ricinus,* again with unknown consequences for its host tick [14]. The recent development of metagenomic approaches on ticks will probably allow the identification of numerous new bacteria species [15], [16].

Even if some of those micro-organisms are not pathogenic for vertebrate hosts, their presence within ticks may have some consequences for the epidemiology of some tick-borne diseases either by acting on vector fitness or through synergic or antagonistic interactions with known pathogens. Recent studies have highlighted the fact that species interactions in a parasite community drive infection risk [17] or that competitive exclusion may be detrimental to one pathogen species in case of co-infections [18]. In the case of blood-feeding insects, symbiotic bacteria having a role in the nutrition of their host have been reported in an increasing number of cases [19], [20]. Moreover, some bacterial symbionts can have direct consequences on the transmission of some pathogens by vectors. For example, the refractoriness of some *Anopheles gambiae* populations from Zambia in transmission of *Plasmodium falciparum* is due to the presence of an *Enterobacter* species in the mosquito's gut [21]. In the mosquito species *Aedes albopictus*, it has been demonstrated that the replication of the Chikungunya virus is modulated by the presence of endosymbiotic *Wolbachia* [22]. *Wolbachia* is also known to induce resistance to dengue virus in *Aedes aegypti* [23]. *Wolbachia* are now also known to interfere with the innate immune system of their arthropod host, hence conferring virus protection in some cases [24].

Wolbachia are endosymbiotic bacteria manipulating host reproductive systems (through cytoplasmic incompatibility or parthenogenesis for example) or as mutualists when they are associated with nematodes [25]. The presence of *Wolbachia* may have direct consequences on the development of other pathogens in the same arthropod vector, and also indirect effects on pathogen epidemiology through impacts on the dynamics and genetic diversity of the vector [26]–[27]. Thanks to the development of molecular tools to look for microorganisms within ticks, *Wolbachia* have been reported from ticks in an increasing number of studies (reviewed in table 1). However, their frequency is usually low (but see [28] where *W. pipientis* reach 7.6% in nymphs) and some studies designed to look for *Wolbachia* in ticks have failed to find any [29]. The consequences of the presence of *Wolbachia* in those ticks are unknown.

**Table 1:** List of studies reporting the presence of *Wolbachia pipientis* associated with ticks.

Tick species	Origin of the tick population	Frequency of ticks harboring *Wolbachia*	Reference
*Ixodes ricinus*	France	7,6% (n = 131)	[28]
*Ixodes ricinus*	Germany	2 clones out of 77, obtained from 27 nymphs	[34]
*Ixodes ricinus*	Germany	0,9% (n = 5424)	[33]
*Ixodes ricinus*	Italy	>1% (two samples of 100 nymphs)	[16]
*Ixodes ricinus*	Morocco	0,5% (n = 221)	[35]
*Ixodes scapularis*	Massachusetts	1 clone out of 106 from 7 nymphs	[46]
*Rhipicephalus microplus*	Texas	?	[15]
*Rhipicephalus sanguineus*	Japan	12,5% (n = 32)	[62]

The purpose of this study was to look for *Wolbachia* in natural populations of *I. ricinus* and to identify their origin within ticks. We discovered that natural populations of the wasp *Ixodiphagus hookeri* (Hymenoptera, Chalcidoidea, Encyrtidae), an endoparasitoid of *Ixodes ricinus*, ,are infected at almost 100% prevalence by *Wolbachia pipientis*. Using PCR primers specific to *I. hookeri* and *W. pipientis* respectively, we showed that in natural tick populations, all individual ticks harboring *Wolbachia* were also parasitized by *I. hookeri*, while ticks that were not parasitized were *Wolbachia-*free. We showed experimentally that some unfed nymphs, exposed to the parasitoids while they were larvae gorging on their vertebrate host, also harbored *Wolbachia*. We hypothesize that the presence of *Wolbachia*, already reported in several studies using 16S PCR primers to identify bacteria within ticks, is due to the presence of *Ixodiphagus* in those ticks, which is externally cryptic (as the parasitized tick nymphs looks healthy until their engorgement that bring the parasitoid eggs out of their diapause). Finally, we discuss the consequences of these findings for our understanding of tick-microbiota and possible consequences for tick-borne diseases epidemiology.

## Results

### The parasitoid *Ixodiphagus hookeri* harbors endosymbiotic *Wolbachia pipientis*

**Figure 1:**
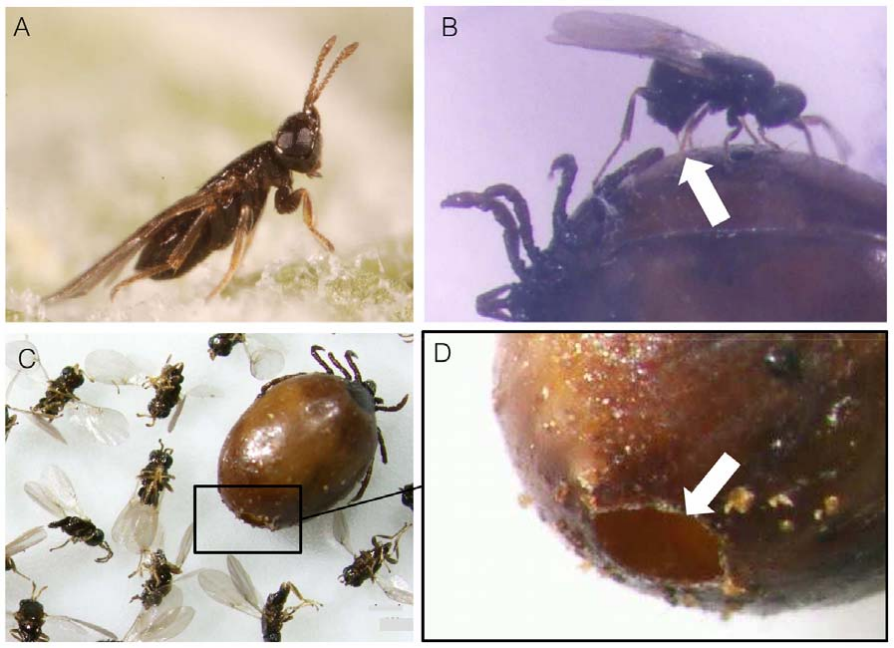
*Ixodiphagus hookeri* (Hymenoptera : Chalcidoidea, Encyrtidae). A Female habitus. B Female ovipositing in an engorged nymphs of *Ixodes ricinus* (ovipositor indicated by the arrow). C Adults of *I. hookeri* around the dead body of an engorged nymph of *I. ricinus*. D Emergence hole from which parasitoids exit the dead body of the engorged nymph.

Based on both (i) engorged nymphs collected on roe-deer and (ii) host-seeking nymphs collected on vegetation that were subsequently engorged in the lab, a total of 54 ticks collected in 4 natural populations were found to be parasitized by *I. hookeri* (Table 2; Figure 1). Variation of parasitism rates among years and sites range from 3,2% to 12,5% in Chizé (tick nymphs collected on roe deer), and from 19,6% to 20% in Gardouch (tick nymphs collected on roe-deer and on vegetation respectively). Among the *I. hookeri* individuals obtained from those 54 ticks, a total of 121 *I. hookeri* individuals (74 females and 47 males) were screened for *Wolbachia pipientis* by PCR using *Wsp* primers. Wolbachia DNA was found in 120 (99,2%) of those individuals (a single wasp male from the Gardouch population was *Wolbachia*-free, even after a second PCR trial).

**Table 2:** Parasitism rates of *Ixodes rcinus* due to *Ixodiphagus hookeri* and presence of *Wolbachia* in the wasps

Tick origin	Sampling site	Sampling date	No. nymphs collected	No. nymphs parasitized by *I. hookeri*	Parasitism rates %	No. nymphs parasitized by *I. hookeri* studied	No. *I. hookeri* individuals studied			No. *I. hookeri* individuals with *Wsp* PCR +
							Female	Male	Total	
Roe deer	Chizé	Feb. 2007	128	16	12,5	0	-	-	-	-
Roe deer	Chizé	Feb. 2008	14	1	7,1	0	-	-	-	-
Roe deer	Chizé	March 2009	221	7	3,2	7	9	6	15	15
Roe deer	Chizé	Feb. 2010	86	6	7	5	5	4	9	9
Roe deer	Chizé	Feb. 2011	205	16	7,8	0	-	-	-	-
Roe deer	Trois-Fontaines	Dec. 2009	20	1	5	1	1	1	2	2
Roe deer	Gardouch	March 2009	40	8	20	8	27	8	35	35
Vegetation	Gardouch	Oct. 2009	101	25	24,8	21	21	18	39	39
Vegetation	Gardouch	April 2010	102	20	19,6	11	7	8	15	14
Vegetation	Fougère	Sept. 2010	6	1	16,7	1	4	2	6	6
Total			923	101	10,9	54	74	47	121	120

### Tick individuals from natural populations parasitized by *I. hookeri* are also harboring *Wolbachia pipientis*

Among 250 unfed nymphs collected in April 2010 in Gardouch, 102 were engorged and the parasitism rate due to *I. hookeri* observed in this natural population was 19,6%. DNA was extracted from 52 of these 250 nymphs, and the presence of both *I. hookeri* and *W. pipientis* DNA was investigated using of hymenopteran *CO1* and *Wolbachia Wsp* specific PCR primers. Ten out of those 52 nymphs (19,2%) were found to harbor both *I. hookeri* and *W. pipientis*. All the 42 ticks that were found without any wasp DNA (i.e. no PCR amplification with the *CO1* primers) were also found without *W. pipientis* (i.e. no PCR amplification with the *Wsp* primers), except for a single tick DNA extract found to be *Wolbachia-*positive but wasp-negative.

### Experimental demonstration that parasitim of *I. ricinus* by *I. hookeri* is associated with the presence of *Wolbachia* in ticks

To demonstrate experimentally that *I. hookeri* is responsible for the presence of *Wolbachia* in some *I. ricinus* individuals, we set up a laboratory rearing of this parasitoid species and exposed some laboratory strain ticks to the parasitoids. Under experimental conditions, parasitism rate was extremely reduced when unfed nymphs were exposed to parasitoids (parasitism rate estimated by the engorgement of unfed nymphs exposed to parasitoids was 2,5% - i.e. 3 nymphs produced wasps of 171 exposed to adult parasitoids and subsequently engorged to reveal parasitism). Parasitoids obtained from those experimentally parasitized ticks again harbored *Wolbachia* as attested by the results of PCR amplification with *Wsp* primers, hence demonstrating experimentally the vertical transmission of *Wolbachia* in *I. hookeri.* Additionnaly, *Wsp* primers were tested successfully on DNA extracted from groups of about 50 *I. hookeri* eggs that were isolated after the dissection of female parasitoid gaster, demonstrating that the parasitoid eggs contain some *Wolbachia*.

Parasitoid rates were higher when nymphs were exposed to parasitoids while they were feeding on gerbils (40,5%; n = 84). However, these engorged nymphs are not the tick stage for which the primers are the most useful (preventing the engorgement of ticks in the lab to determine the parasitism rate), i.e. the unfed nymphs. To produce unfed nymphs in the lab that harbor *I. hookeri*, we exposed some tick larvae to parasitoids during engorgement on gerbils. Previous studies [30], [31] have shown that such tick larvae produce living nymphs that harbor parasitoid eggs and only the engorgement of those parasitized nymphs brings the wasp eggs out of diapause, allowing the parasitoids to emerge from those engorged nymphs. The set of nymphs exposed to parasitoids during engorgement was divided in two. Half were used to estimate the parasitism rate due to *I. hookeri*. Two of 33 nymphs produced some parasitoids after engorgement, corresponding to a parasitism rate of 6%. The second half was used to look for *Wolbachia* using the *Wsp* primers; 8 of the 106 unfed nymphs (5,7%) harbored *Wolbachia*. Two out of those 8 ticks harboring *Wolbachia* were also positive with the CO1 primers, confirming that nymphs containing *Wolbachia* also contained *I. hookeri* eggs. We interpreted the lack of amplification with the CO1 primers for 6 out of those 8 ticks as due to lower sensitivity of the CO1 primers and the low quantity of *I. hookeri* DNA expected in a parasitized nymph (containing 5 to 10 eggs). PCR screening with appropriate primers showed none of the 32 unfed nymphs used as a negative control (corresponding to larvae that were not exposed to parasitoids during their engorgement) to contain *Wolbachia* or *I. hookeri* DNA.

### *Wolbachia pipientis* associated with *I. hookeri* belongs to a *Wolbachia* clade found in hymoneptera or other insects

Sequence data for *Wsp* and *ftsZ* genes of *Wolbachia* found in *I. hookeri* from ticks was used to determine whether these bacteria belong to a clade already associated with other insects (including parasitoids), or instead fall within *Wolbachia* groupings associated with ticks (Acari) or other Arachnida and Chelicerata. Six *Wsp* sequences (ranging from 515 to 379 pb) were obtained from PCR products based on *I. hookeri* individuals from the Gardouch and Chizé populations. The alignment revealed no polymorphism (in the 351 pb common to all 6 sequences or in the other part of the alignment). A consensus sequence of 533 pb was generated (515 pb from the longest sequence, to which 18 pb were added from another sequence) and compared to *Wsp* sequences already deposited in GenBank with BLAST. Over those 533 pb, this consensus sequences has 100% identity with a *Wolbachia* associated with *I. ricinus* (accession n° EF219192.1 from a dutch population; Huigens,M.E. et al. unpublished) and 99% identity with 16 *Wsp* sequences of *Wolbachia* associated with insects and particularly Hymenoptera (2 with another parasitoid chalcid – *Spalangia cameroni* [Hymenoptera: Pteromalidae], 10 with the parasitoid *Asobara tabida* [Hymenoptera : Braconidae], 1 with the parasitoid *Trichopria drosophilae* [Hymenoptera: Diapriidae], 2 with the moths *Dichocrocis punctiferalis* and *Ostrinia furnacalis* [Lepidoptera : Crambidae] respectively and 1 with the bug *Elasmucha signoreti* [Heteroptera : Acanthosomatidae]).

**Figure 2:**
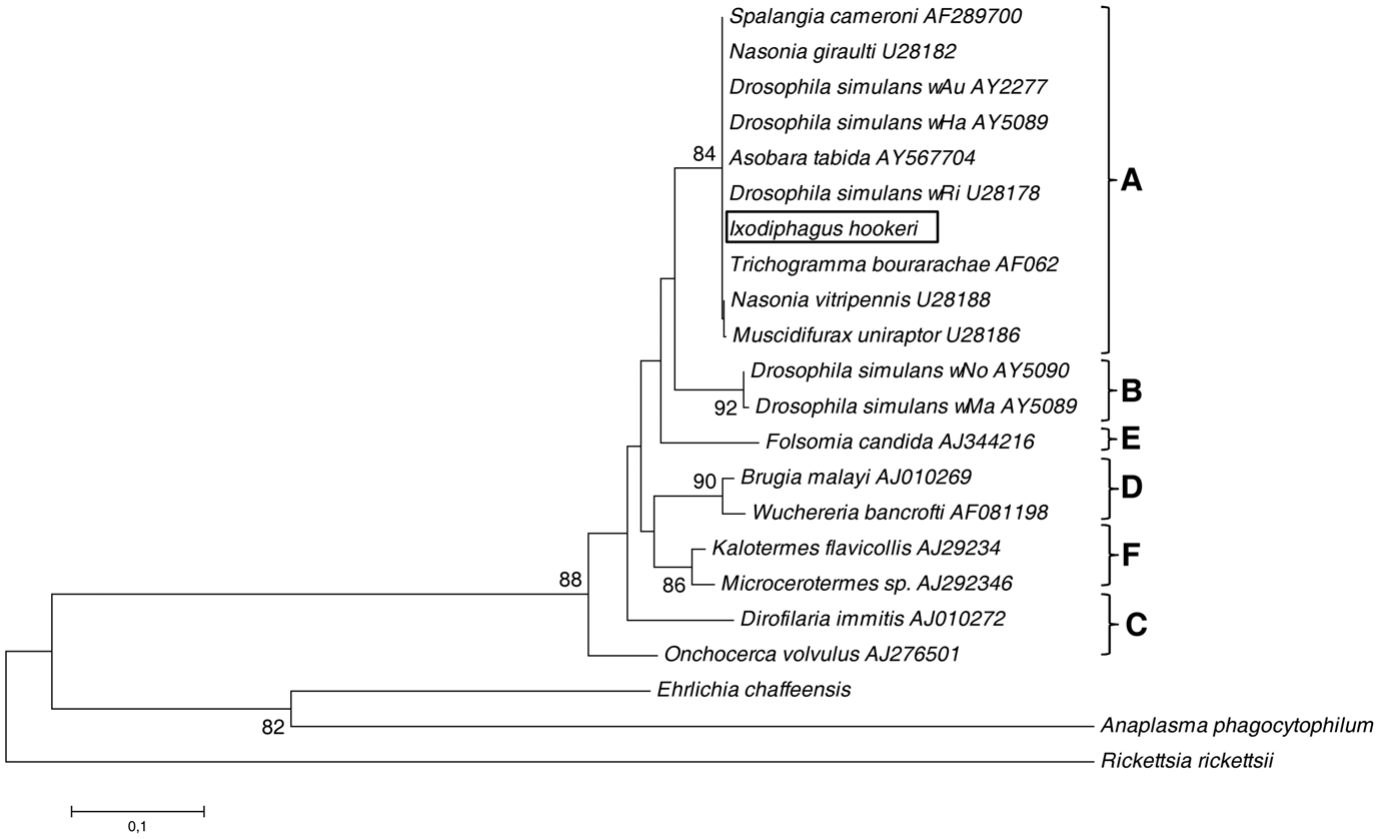
Phylogenetic tree of *Wolbachia pipientis*. A Neighbor-Joining tree based on the the *Ftsz* sequence obtained with MEGA5 using the Maximum Composite Likelihood distance. Only bootstrap values greater than 80% are shown. Letters A to F (at the right of the name of the host from which the *Wolbachia* was sequenced and of its accession number) refer to the *Wolbachia* supergroups already described [58].

Because the *Wsp* gene is known to exhibit a high recombination rate [32], we also sequenced the *Ftsz* gene to identify the *Wolbachia* supergroup to which the *Wolbachia* associated with *I. hookeri* belong. The *FtsZ* sequence (947 bp long) was obtained from a wasp individual from the Gardouch population. In the phylogenetic tree (Figure 2), this sequence clearly belongs to supergroup A (with only one difference with the sequence found in the *Wolbachia* associated with other hymenopteran parasitoids such as *Spalangia cameroni*, *Trichogramma bourarachae* (Hymenoptera; Chalcidoidea; Trichogrammatidae), *Asobara tabida*, and the fruitfly *Drosophila simulans*. The *Ftsz* and the *Wsp* sequences have a 100% similarity with the allele ftsz-3 (435 bp long) and Wsp-40 (493 bp long) respectively in the *Wolbachia* MLST database (*Wolbachia* MLST website: http:pubmlst.org/wolbachia).

## Discussion

### Presence of *I. hookeri* in *I. ricinus* nymphs is correlated with presence of *W. pipientis* in ticks

By the use of specific PCR primers for the parasitoid *I. hookeri* and for the endosymbiotic bacteria *W. pipientis*, we demonstrated that almost all (99,2%) of the *I. hookeri* individuals investigated, emerging from 5 different natural french populations of the tick *I. ricinus*, harbored *W. pipientis.* The analysis of a natural tick population suffering from high parasitism due to *I. hookeri* (evaluated at 20%, based on the engorgement in the lab of nymphs collected on the vegetation) revealed that all the DNA extracts of nymph ticks showing evidence of the presence of *I. hookeri* (based on *CO1* hymenopteran specific primers) also harbored *W. pipientis* (based on *Wsp Wolbachia* specific primers), while all but one of the tick DNA extracts without any evidence of *I. hookeri* were also *Wolbachia-*free. We also showed experimentally that unfed nymphs developing from larvae exposed to wasps while gorging on their host, also harbored *Wolbachia*. Moreover, the *Wolbachia* sequence of the *Wsp* and *Ftsz* genes associated with *I. ricinus* were identical or very similar to *Wolbachia* sequences already reported as associated with parasitoid hymenoptera, including other chalcids.

Based on these observations, we suggest that the presence of *W. pipientis* in *I. ricinus*, already reported by several authors (but, to date, without any hypothesis to explain the presence of this bacterium in ticks), is due to the parasitism of those ticks by the hymenopteran endoparasitoid *I. hookeri*. Because of the koinobiontic development of those parasitoids, the presence of the parasitoids in nymphs cannot be detected visually prior to their engorgement (bringing the parasitoid eggs out of diapause). In all of these studies (Table 1), *Wolbachia* has been identified from the sequencing of 16S rRNA gene amplicons. Ticks were sampled from natural populations (and not laboratory reared) and thus these ticks could have been exposed to *I. hookeri* parasitoids. Moreover, our hypothesis is corroborated by the fact that most of these studies are based on tick DNA extracts obtained from nymphs [16], [33], [34], the tick stage most attacked by the parasitoids [28]. Two other studies have reported the presence of *W. pipientis* from adults of *I. ricinus*, but at a much lower frequency (*i.e* 1,0% (n = 98) compared to 27,3% in nymphs (n = 33) in a French population [28]; 0,45% (n = 221) in a Moroccan population; [35]; see also [15] for the report of *W. pipientis* associated with adults of *Rhipicephalus (Boophilus) microplus*). Although emergence of *I. hookeri* has never been reported from engorged *I. ricinus* females [36] (but oviposition on adults occurs in the lab on adult *Rhipicephalus sanguineus* [37]), the presence of *W. pipientis* in a few adult ticks may still be explained by parasitism of nymphs by *I.hookeri.* Indeed, it is likely that not all ovipositions of *I. hookeri* result in successful development of parasitoids. In particular, the innate immune system of ticks may be able to kill some parasitoid eggs (by the encapsulation of parasitoid eggs resulting in its melanization and subsequent death) that could prevent the development of parasitoids but explain the presence of *W. pipientis* DNA in tick adults. The fact that *W. pipientis* has not been found in some studies that have looked for it in *I. ricinus* [29], [38] can also be explained by the absence of parasitoid in those ticks.

### Use of *Wolbachia* specific primers to estimate parasitism rates due to *I. hookeri*

In subsequent studies, *Wolbachia-*specific *Wsp* primers could be used to determine parasitism rates due to *I. hookeri* in natural populations of *I. ricinus* using host-seeking nymphs (and thus avoiding the time-consuming engorgement of nymphs in the lab to determine parasitism rates). Because parasitoid diapause occurs at the egg stage [39], the amount of *I. hookeri* and *Wolbachia* DNA in unfed *I. ricinus* nymphs is probably reduced (5 to 10 eggs per ticks considering the mean number of parasitoid adults emerging from an engorged nymph; 6,5 according to [40]; n = 54). Fortunately, the use of mitochondrial primers (the DNA mitochondrial molecules being present in numerous copies in each single egg cell) is more sensitive to report the presence of *I. hookeri* than nuclear markers. However, the use of *Wsp* primers seems to be even more sensitive to report the presence of *I. hookeri* in ticks than the specific mitochondrial *CO1* primers (c.f. the few cases where tick extracts were positive with the *Wsp* and not the *CO1* primers). This may be due to a high number of *Wolbachia* bacteria in *I. hookeri* eggs (for example, between 116 and 292 bacteria per egg in *Nasiona vitripennis* [41].

Our results show that parasitism rates due to *I. hookeri* in natural population of *I. ricinus* can reach at least 20%, inflicting significant mortality on tick populations. This explains why *I. hookeri* was considered as a possible biological control agent to reduce tick populations, soon after its original description [42]. The higher parasitism rates observed in Gardouch may be associated with the higher roe deer density (as already observed in the USA for *Odocoileus virginiae, Ixodes scapularis* and *I. hookeri* [43]) maintained in a 14 hectare fenced area. In Chizé, the parasitism rates are relatively stable across years (again fenced, but covering a much larger area of 2580 hectares). However, the month of tick sampling (February), constrained by roe-deer capture, may not be representative of the parasitism rates of *I. ricinus* during other months.

### Consequences of the presence of *Wolbachia* on tick and tick-borne diseases

The phenotypic consequences of *Wolbachia* infection for their hosts are highly diverse and new impacts are regularly discovered. In filarial nematodes, these bacteria are mutualistic and required for the development and reproduction of their host. In arthropods, they have first been shown to alter their host reproduction through various mechanisms (cytoplasmic incompatibility, parthenogenesis, male-killing, feminization…) but a growing number of studies show that they also have some impacts on the immune response of their host (with some consequences for resistance against parasitoids, or on pathogen agents transmission in the case of arthropod vectors; [44]) or may even be nutritional mutualists in blood feeding bugs [20]. Previous reports of *W. pipientis* in ticks have only mentioned the known consequences of *Wolbachia* on nematodes or insects [33] or suggested their possible role as mutualistic endosymbionts of ticks [45] or as pathogenic agents [35]. Our finding that *Wolbachia* is associated with *I. hookeri* rather than with *I. ricinus per se* and that *Wolbachia* is thus not (or only very rarely) vertically transmitted to tick offspring (otherwise, the prevalence of *Wolbachia* in ticks would be much higher; anyway, most ticks parasitized by *I. hookeri* and thus containing *W. pipientis* are expected to die, once they will engorge) suggests that any consequences of *Wolbachia* infection on *I. ricinus* reproduction are without object. Additionnal investigations must be conducted to identify the consequences of *Wolbachia* on *I. hookeri*. Although the presence of *Wolbachia* in ticks may be considered a “dead-end” for the bacteria, it may have had some consequences on other microorganisms harbored by ticks. For example, a *Wolbachia* gene has been identified by Ishmael et al. within a plasmid of a *Rickettsia* species found in *Ixodes scapularis* (the “*Rickettsia* endosymbionts of *I. scapularis*” identified by Nodae et al. [45] and Benson et al.[46] and reported in 100% of the engorged females studied by [47]). The authors suggest that lateral gene transfer between these two obligate intracellular species has occurred. The 44 *Wolbachia* traces found in the *I. scapularis* genome [48] were finally due to the presence of these *Rickettsia* endosymbionts within the *I. scapularis* trace analysed [49].

Beside *W. pipientis*, a negative interaction between some pathogen agents (*i.e.* the Spirochaete *Borrelia burgdorferi* and the Apicomplexa *Babesia microti*) and *I. hookeri* in north American populations of *I. scapularis* has been reported [50]. Although this study was conducted prior to the development of molecular techniques to detect those micro-organisms within ticks, the authors observed that the prevalence of those human pathogens in host-seeking ticks collected in wasp-infested sites is nearly 40% lower than that found in other sites and that spirochaetes never infected wasp-infected ticks and few wasp-infected ticks were concurently infected by *B. microti*. The authors considered that “wasps may render the tick inhospitable to both pathogens. Our finding that *I. hookeri* harbors *W. pipientis* could provide an explanation for those observations : the bacteria may induce an immune reaction in the ticks that could have a negative impact on other micro-organism hosted by ticks (see for example [44]).

To conclude, in prevision to the growing number of studies that will use a metagenomic approach to identify micro-organisms within ticks [15], we would like to highlight the fact that ticks are also the hosts of macroparasites (including wasps such as *I. hookeri*, but also nematodes such as *Cercopithafilaria rugosicauda* associated with *I. ricinus*) that can harbor their own microbiome. Thus, the study of tick-borne diseases involves more than a tripartite system involving vertebrates, arthropod vectors and pathogen agents but also involves other organisms, pleading for a community ecology approach on these complex systems.

## Materials and Methods

### Tick and insect populations studied

#### Ixodes ricinus

The tick *Ixodes ricinus* is the most common european tick species, found mainly in forests, but also hedges or moors. Its 2–4 year lifecycle includes three stages (larva, nymph and adults), with each stage needing to take a blood meal on a vertebrate host (a mammal, bird or lizard) for its development. After each blood meal, the engorged ticks fall from the host to the ground, where they will metamorphose to the next stage. Questing (host-seeking) ticks will then waiting for a host by staying on the top of the vegetation.

Natural populations of *Ixodes ricinus* were collected from 5 different locations in France, either by collecting engorged tick nymphs directly on roe-deer (Réserve Biologique Intégrale de Chizé - 46° 7′18.89″N, 0°25′3.72″O -, Trois Fontaines - 48°41′46.14″N, 4°55′30.31″E - and Gardouch - 43° 23′ 27.6″ N, 01° 40′ 52.0″ E), or by collecting unfed questing tick nymphs on the vegetation using the standard “flagging method” (Gardouch and Fougère - 47°36′25.99″N, 0° 5′54.84″O -) from 2007 to 2010. Additional tick nymphs from our laboratory strain (originally collected in Belle-Ile; [51]) were used as a negative control.

#### Ixodiphagus hookeri

With 8 species described [52], *Ixodiphagus* (Hymenoptera, Chalcidoidea, Encyrtidae) is the single parasitoid genus of 80 000 parasitoid species described [53] known to parasitize ticks. In Europe, only *I. hookeri* has been reported, where it is mainly associated with *Ixodes ricinus.* Although the lifecycle of this parasitoid is not completely known (especially the stages during winter time; [40]), the parasitoids are only known to emerge from engorged nymphs. The females of *I. hookeri* oviposit 5–10 eggs into tick nymphs (or eventually in tick larvae). The parasitoid does not kill its host during oviposition (corresponding to the koinobiontic strategy) and the tick nymphs look healthy until their engorgement. Once the tick is engorged, the diapause of the wasp eggs is broken and the wasp larvae begin their development within the engorged tick. The tick will finally die and the adult parasitoids dig a hole at the posterior end of the tick to emerge, 1–2 months after oviposition. The adult wasps then mate and the females look for ticks to lay their eggs before to die 2 or 3 days after emergence.

### Estimation of the parasitism rates due to *I. hookeri* in natural populations of *I. ricinus*

Parasitism rates due to *I. hookeri* in natural populations of *I. ricinus* were estimated both from engorged ticks collected on roe-deer and from questing ticks collected on the vegetation. Unfed tick nymphs were engorged on gerbils, after having been laid on the fur with a paintbrush and recovered 3 to 7 days after. All engorged tick nymphs were then placed in individual tubes and kept at a humidity of 80%. Emergence of parasitoids from engorged nymphs was checked weekly. The adults obtained were sexed under a steromicroscope using the key provided in Quaraishi 1958 [54]. Parasitism rates were calculated by taking into account only engorged nymphs producing either adult ticks or parasitoids (engorged nymphs that die without producing adult ticks or wasps were not included).

### Exposition of *I. ricinus* to the parasitism by *I. hookeri* in laboratory experiments

Unfed nymph ticks from our laboratory rearing (without *I. hookeri* and shown by *Wsp* PCR to be *Wolbachia*-free), were exposed to parasitoids in sets of 25 (2 flasks), 50 (2 flasks)or 100 (3 flasks) individuals in a tissue culture cell flask (25 cm^3^) with filter screw cap (TRP). Several freshly emerged adult parasitoids (both females and males; from 4 to 10; mean of 7 wasps per flask) were added to each flask for 2 or 3 days until parasitoid death. In other experiments, tick nymphs or larvae were exposed to parasitoids while they were gorging on gerbils. To that aim, several freshly emerged wasps (from 4 to 7, mean of 5,8 per cage) were introduced to the cage containing the gerbil and the ticks that were deposited on the gerbil 4 days before.

### DNA extraction

Individual ticks or wasps, killed at −80°C or conserved in alcohol, were put in one well (in 2 ml Deepwell plate; NUNC) with 2 pre-sterilised glass beads of 3 mm diameter and one of 5 mm. After 15 minutes at −80°C, they were mechanically crushed by using the Qiagen Tissue Lyser for 3 minutes at a frequency of 15 Hz. DNA was extracted using the NucleoMag 96 Tissue kit (Macherey-Nagel). DNA was eluted with 50 µl buffer for ticks and 25 µl for wasps and conserved at 4°C until PCR was performed.

### PCR amplification and sequencing

To amplify the *wsp* gene of Wolbachia, we used the *wsp-*81F and *wsp-*691R primers [55] amplifying a DNA fragment of 590-632 pb. PCRs were conducted in 20 µl reaction volumes with 7,8 µl H_2_O (EPPI sterile), 4 µl 5X Buffer, 1 µl forward and reverse primer (10 µM), 2 µl dNTP (2mM), 0,2 µl of Phusion High-Fidelity Taq (New England Biolabs) and 4 µl of DNA extract. The amplification program is the following: 98°C for 30 seconds, then 29 cycles at 98°C for 45 seconds, 55°C for 45 seconds and 72°C for 60 seconds and final elongation at 72°C for 10 minutes. The PCR product obtained was sequenced using PCR primers as sequencing primers (accession number : JQ315223).

To amplify the *ftsZ* gene of Wolbachia, we used the *ftsZf1* and *ftsZr1* primers [56]. They amplified a DNA fragment of 1043–1055 pb. PCRs were conducted in 25 µl reaction volumes with 19,3 µl H_2_O (EPPI sterile), 2,5 µl 10X Buffer, 0,350 µl forward and reverse primer (20 µM), 0,5 µl dNTP (10mM), 0,75 µl MgCl2, 0,25 µl of Eurobio Taq and 1 µl of DNA extract. The amplification program is the following: 94°C for 60 seconds, 55°C for 60 seconds, 72°C for 3 minutes, then 35 cycles at 94°C for 15 seconds, 55°C for 60 seconds and 72°C for 3 minutes and final elongation with a cycle at 94°C for 15 seconds, 55°C for 60 seconds, 72°C for 10 minutes. The PCR product obtained was sequenced using PCR primers as sequencing primers (accession number : JQ315224).

To design specific primers for the *CO1* gene of *I. hookeri* (that do not amplify *I. ricinus* DNA), we first used the primers 1775-Co1-F, and 2773-Co1-R, already described for other Chalcidoidea [57]. From the *I. hookeri* sequence obtained (accession number JQ315225), we designed two new primers 401-F (5′-TTTAGAATATTTATTGATTCAGGGACT-3′) and 44-R (5′-CTCCTGCTAAAACTGGTAAAGATAAT-3′) that amplify a 268 bp fragment. The non-amplification of *I. ricinus* with those primers specific to *I. hookeri* was confirmed by PCR.

PCRs were conducted in 20 µl reaction volumes with 9,2 µl H_2_O (EPPI sterile), 2 µl 10X Buffer (Eurobio), 1 µl forward and reverse primer (10 µM), 2 µl dNTP (2mM) and 0,6 µl MgCl_2_ (50 mM, Eurobio), 0,2 µl of Eurobio Taq and 4 µl of DNA. The amplification program is a touchdown program : 92°C for 2 minutes, then 92°C for 45 seconds, 53°C to 47°C for 45 seconds and 72°C for 60 seconds, then 29 cycles at 92°C for 45 seconds, 47°C for 45 seconds and 72°C for 60 seconds and final elongation at 72°C for 10 minutes.

### Alignment and Phylogenetic analysis

To identify the *Wolbachia* supergroup of the *Wolbachia* sequence associated to *I. hookeri,* we downloaded *Ftsz* sequences from the *Wolbachia* supergroups A, B, C, D, E, F [58] to which we have added several *Ftsz* sequences with high query coverage and percentage of identity using BLAST. The sequences of *Anaplasma phagocytophilum*, *Rickettsia rickettsi* and *Ehrlichia chaffeeensis* were also added as outgroups and to illustrate the divergence between those well-known pathogen agents transmitted by ticks and *W. pipientis.* The alignment of the *Ftsz* sequences was conducted with CLUSTAL W2 [59]. Phylogenetic analyses were conducted with MEGA5 [60]. A Neighbor-Joining tree was built with the Maximum Composite Likelihood distance [61], and a complete deletion option. Bootstrap values were estimated using 10,000 replications.

### Ethical Statements

All experiments were carried out at Oniris (Ecole Nationale Vétérinaire, Agroalimentaire, et de l′Alimentation Nantes Atlantique), adhering to local (Pays de la Loire Ethics Committee) and national guidelines and laws of experimental work with laboratory animals who has approved the protocol used in this study (permit number n° 44266).
